# Integrated edge-to-exascale workflow for real-time steering in neutron scattering experiments

**DOI:** 10.1063/4.0000279

**Published:** 2024-12-24

**Authors:** Junqi Yin, Viktor Reshniak, Siyan Liu, Guannan Zhang, Xiaoping Wang, Zhongcan Xiao, Zachary Morgan, Sylwia Pawledzio, Thomas Proffen, Christina Hoffmann, Huibo Cao, Bryan C. Chakoumakos, Yaohua Liu

**Affiliations:** 1 National Center for Computational Sciences, Oak Ridge National Laboratory, Oak Ridge, Tennessee 37831, USA; 2 Computer Science and Mathematics Division, Oak Ridge National Laboratory, Oak Ridge, Tennessee 37831, USA; 3 Computational Science and Engineering Division, Oak Ridge National Laboratory, Oak Ridge, Tennessee 37831, USA; 4 Neutron Scattering Division, Oak Ridge National Laboratory, Oak Ridge, Tennessee 37831, USA; 5 Second Target Station, Oak Ridge National Laboratory, Oak Ridge, Tennessee 37831, USA

## Abstract

We introduce a computational framework that integrates artificial intelligence (AI), machine learning, and high-performance computing to enable real-time steering of neutron scattering experiments using an edge-to-exascale workflow. Focusing on time-of-flight neutron event data at the Spallation Neutron Source, our approach combines temporal processing of four-dimensional neutron event data with predictive modeling for multidimensional crystallography. At the core of this workflow is the Temporal Fusion Transformer model, which provides voxel-level precision in predicting 3D neutron scattering patterns. The system incorporates edge computing for rapid data preprocessing and exascale computing via the Frontier supercomputer for large-scale AI model training, enabling adaptive, data-driven decisions during experiments. This framework optimizes neutron beam time, improves experimental accuracy, and lays the foundation for automation in neutron scattering. Although real-time experiment steering is still in the proof-of-concept stage, the demonstrated potential of this system offers a substantial reduction in data processing time from hours to minutes via distributed training, and significant improvements in model accuracy, setting the stage for widespread adoption across neutron scattering facilities and more efficient exploration of complex material systems.

## I. INTRODUCTION

Neutron scattering is a powerful technique for studying structural and dynamic properties, including both lattice and magnetism of materials.^1^ At the Spallation Neutron Source (SNS), Oak Ridge National Laboratory (ORNL), neutron time-of-flight (TOF) scattering experiments generate rich, high-dimensional data in event mode, allowing for unprecedented exploration of material properties in multidimensional parameter spaces.^2^ Event-mode data capture individual neutron events rather than aggregating them into histogrammed spectra.^3^ Although these individual events are subsequently rebinned into traditional histogram formats (such as intensity vs *d*-spacing with or without energy resolution), the original event-based data are reserved in NeXus format.^4^ Event-based neutron data open doors to more advanced measurements with continuously varying parameters, which are asynchronous to the neutron sources.^5^ However, these experiments have traditionally relied on post-experiment data analysis, which significantly limits feedback and reduces the ability to adjust experimental conditions in real time. This gap is particularly critical in single-crystal neutron diffraction, where complex scattering patterns often demand immediate adjustments to optimize data collection.^6^ To address these challenges, we have developed a scalable workflow for real-time steering in neutron scattering that identifies data stability to prevent wasted beam time and optimize resources using machine learning. In the context of neutron scattering, real-time experiment steering refers to the ability to dynamically guide and adjust the experimental parameters and data collection strategy during the experiment, rather than after data collection has completed.

The four-dimensional (4D) temporal event data generated in neutron scattering experiments at SNS are complex and computationally intensive to analyze, making it difficult for traditional methods to process the data fast enough to inform real-time decisions. This calls for scalable computational solutions and advanced predictive models capable of handling the high-dimensionality of event-mode neutron data. The integration of edge and exascale computing systems is crucial in addressing these challenges. Edge computing here refers to on-site systems, such as EPICS (Experimental Physics and Industrial Control System)^7^ for data acquisition and ADARA (Accelerating Data Acquisition, Reduction, and Analysis)^8^ for streaming, that control and manage real-time neutron data. On the other hand, exascale computing, harnessing the immense computational power of Frontier,^9^ the first exascale supercomputer, at the Oak Ridge Leadership Computing Facility (OLCF),^10^ has great potential to enable the rapid processing and prediction of neutron scattering patterns using advanced machine learning/artificial intelligence (ML/AI) models. This synergy between real-time data acquisition and large-scale predictive analysis moves us closer to achieving real-time experiment steering.

This work presents a proof-of-concept demonstration using measured event data from the SNS TOPAZ Beamline,^11^ showcasing a scalable edge-to-exascale workflow for real-time neutron scattering experiment steering. The goal is to create an efficient system that allows real-time decisions about experimental parameters based on live data, optimizing both data collection and the scientific discovery process. At the heart of this workflow is the Temporal Fusion Transformer (TFT), a deep learning model designed for time series forecasting, combining the strengths of recurrent neural networks (RNNs) and transformer architectures with multihead attention to effectively capture both short-term dependencies and long-range patterns in time series data.^12^ Our TFT model integrates diverse input features such as neutron count rates and time-of-flight data to predict neutron scattering patterns at voxel-level precision. This model requires the computational power of exascale high-performance computing (HPC) resources for training, due to its complex architecture with multi-headed attention mechanisms and interpretable variable selection networks. Frontier, the exascale supercomputer at OLCF, provides the resources needed to train the model on vast and complex 4D temporal datasets,^13^ ensuring that the predictions are accurate and adaptable to real-time neutron data.

Here, we extend our previous work by integrating edge and exascale computing into the neutron scattering workflow that allows for dynamic, real-time feedback. This iterative workflow enables the experimental conditions to be fine-tuned during data collection, ensuring that the most relevant data are captured as early as possible. While this study used stored data, where neutron event data in NeXus format serve as a proof-of-principle for the workflow, the edge-to-exascale workflow establishes the foundation for future integration of live data streams via the SNS ADARA system.^8^ ADARA's ability to provide live data streaming complements NeXus files, which contain saved event and metadata for detailed post-experiment analysis. Ultimately, this system aims to enable real-time steering of experiments, which will be particularly beneficial in complex studies such as multidimensional crystallography, where adaptive feedback can accelerate discovery.

The following sections outline the development of the transformer-based model, the optimization of distributed training across edge and exascale systems, and the validation of this approach on the SNS TOPAZ beamline. By integrating AI/ML with HPC in neutron scattering, this work marks a paradigm shift toward more efficient, automated experimental workflows, aligning with the Department of Energy's vision for Integrated Research Infrastructure (IRI),^14^ which seeks to couple experimental facilities with HPC resources to drive scientific innovation. Through this demonstration of edge-to-exascale integration, we provide a roadmap for future AI-driven steering systems, paving the way for more efficient neutron scattering experiments.

## II. METHODOLOGY

The edge-to-exascale framework includes three core components: (1) a transformer-based model designed specifically for neutron event data, (2) a communication optimization algorithm for distributed training on exascale computing systems, and (3) a stream learning system for edge-enabled, real-time feedback. This combination of technologies not only enhances scientific discovery but also reduces operational costs by improving experiment efficiency.

### A. Edge-to-exascale integration for real-time experiment steering

The approach outlined here demonstrates the workflow for real-time steering of neutron scattering experiments at the SNS, emphasizing optimization of communication between the SNS data cluster and the Frontier exascale system at OLCF. The framework integrates critical components, including EPICS for instrument control,^7^ ADARA for live data streaming,^8^ and transformer-based AI models (such as the Temporal Fusion Transformer, TFT), to predict 4D temporal neutron scattering pattern.^13^ Utilizing edge computing on a DGX workstation at SNS enables real-time experiment steering based on live data streams, which allows dynamic experiment adjustments, enhancing both responsiveness and decision-making.

ADARA,^8^ the data streaming infrastructure currently deployed across all SNS beamlines and implemented at HFIR beamlines, provides live streaming of time-of-flight neutron event data. Its high-performance publish-subscribe model ensures efficient, low-latency data distribution, enabling real-time analysis and feedback during experiments. This capability is critical for integrating edge-to-exascale workflows, supporting dynamic decision-making and optimizing experimental conditions.

**FIG. 1.**
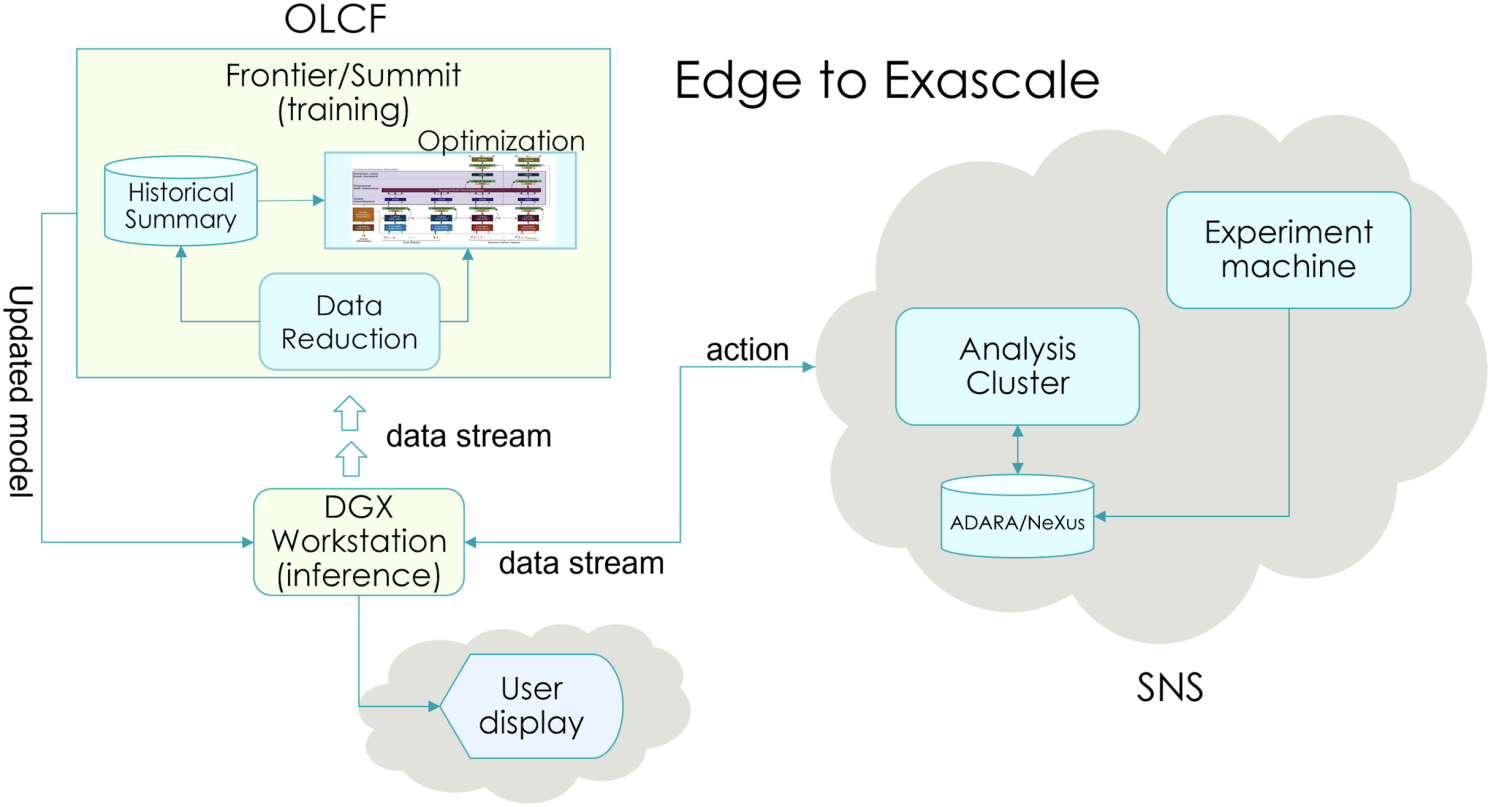
Edge-to-exascale integration approach between OLCF and SNS. Experimental data from SNS are processed on the analysis cluster, followed by data reduction and optimization on Frontier/Summit at OLCF for training at exascale. Inference is performed on a DGX workstation, with results displayed in real time for users, enabling seamless feedback and integration between edge devices and exascale systems for experiment steering.

The key outcome of this work is a scalable application for real-time steering, deployed across the SNS and Frontier systems. Figure 1 illustrates the edge-to-exascale integration approach between OLCF and SNS, designed with portability in mind for seamless integration into ORNL's Interconnected Science Ecosystem (INTERSECT).^15^ Our main contribution is the development of the AI-assisted steering workflow and the demonstration of its effectiveness for a real application. The overview of the workflow steps is provided in Scheme 1.

Scheme 1. Workflow steps:

1. Resources initialization

 a. Start the listener service to monitor the data stream

 b. Allocate the computing resources on Frontier

2. Data preprocessing

 a. Bin and slice data

 b. Transfer the reduced data to Frontier

3. Model training

 a. Start the distributed model training once enough data are collected

 b. Save the latest model checkpoint

4. Real-time feedback

 a. Reconstruct data at future time steps with model inference

 b. Evaluate prediction error

 c. Adjust experiment parameters accordingly

To optimize communication for distributed training on the Frontier exascale system, we implemented a workflow that pulls live data in user-specified time-intervals from the SNS data analysis cluster to OLCF for real-time processing. As new data are generated, a monitoring process on the DGX workstation at SNS tracks file changes and synchronizes reduced NumPy arrays in batches with the Frontier system via a 100 Gigabit Ethernet connection. Once a predefined number of data slices is collected, job scripts are automatically submitted to Frontier for training the TFT model and predicting future neutron scattering events. The training process and model predictions are evaluated based on predefined convergence criteria, such as maintaining a relative error below 5%, which ensures both efficiency and accuracy in real-time experiment steering. By leveraging the high interconnect bandwidth and parallel processing capabilities of Frontier's MI250X GPUs, we achieved seamless integration of HPC resources into the experimental workflow and demonstrated significant improvements in beamline productivity and experiment steering efficiency.

### B. Workflow implementation

Considering SNS (data analysis cluster) and OLCF are situated in different network zones, i.e., open and moderate security, respectively, the proof-of-the-concept workflow is implemented in a data-pulling way from OLCF, where a main process is monitoring the file changes at the remote data source and sync up new data slices.

**FIG. 2.**
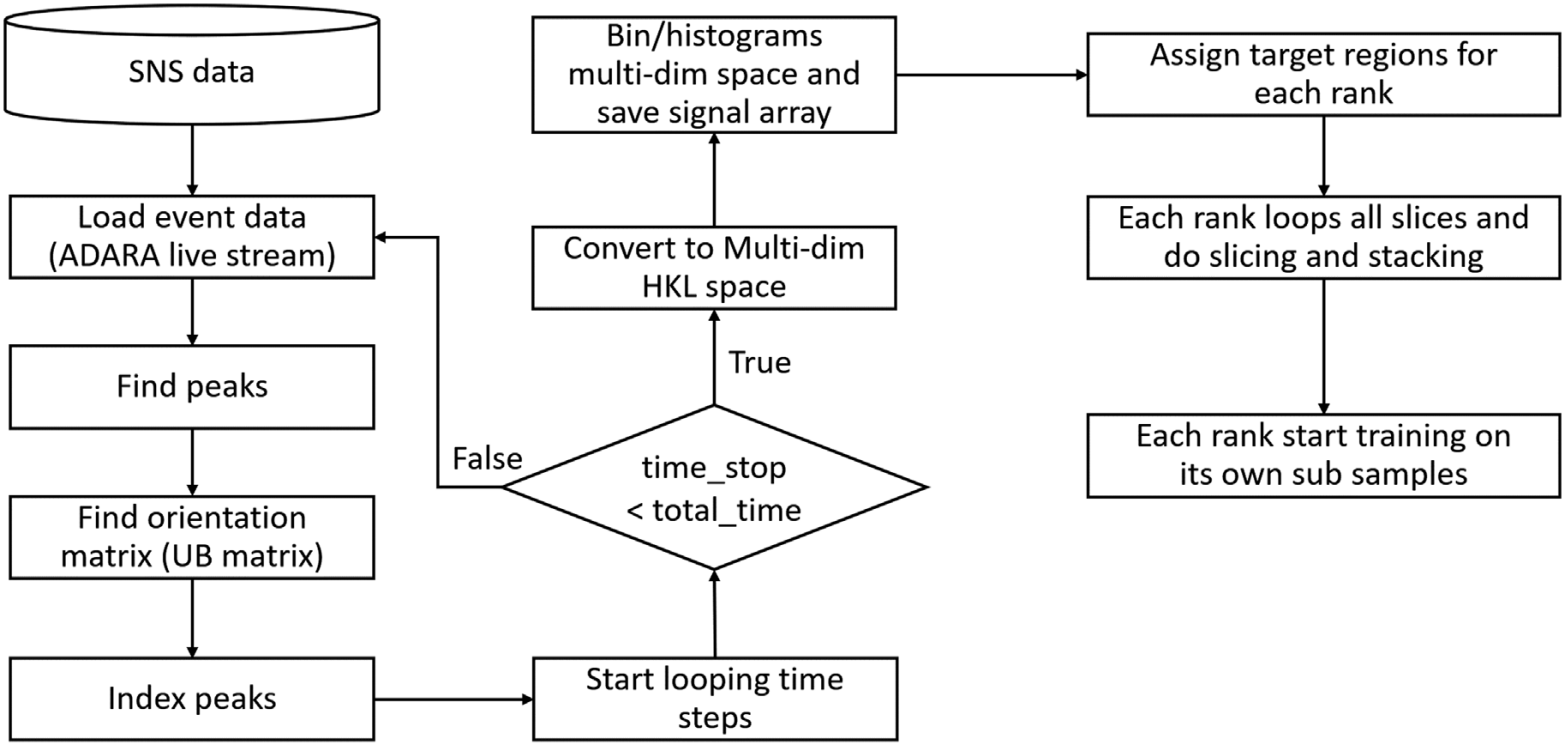
Workflow diagram illustrating the distributed data processing and model training for neutron event data. Live streaming from SNS via ADARA is followed by peak detection and indexing. Time slices are converted to multi-dimensional HKL space and distributed across compute ranks for Temporal Fusion Transformer (TFT) model training on subsamples. The loop continues until all time steps are processed, enabling real-time analysis and experiment steering.

The implementation of the stream learning framework for edge-enabled, real-time feedback involves the continuous processing of neutron event data (either by loading measured data or live data stream from ADRA) in a loop (as illustrated in Fig. 2). The flow chart outlines the workflow for processing neutron event data and conducting distributed training using edge computing on a DGX box at SNS and Frontier at ORNL. Initially, the event data are streamed live via ADARA and binned into a 4D histogram based on measurement time, detector pixel data. After unit conversion and pixel mapping, the event data are compressed and sliced into smaller, manageable subsets, often based on regions of interest such as specific TOF windows or spatial coordinates. For single crystal neutron diffraction, this is followed by converting the data into multi-dimensional space and finding peaks using the available algorithms in Mantid.^16^ The orientation matrix (UB matrix)^17^ is determined next, and the peaks are indexed.^11,18^

Once the initial processing is complete, the workflow enters a time-loop. In this loop, the large dataset is divided into manageable time slices in 3D HKL space and distributed across multiple ranks for efficient processing. Each rank slices the 4D data along the time axis, stacks these slices to create training datasets, and begins training TFT model on the assigned sub-samples. As the process progresses, predictions from all ranks are aggregated, and optional steps allow for visualization, analysis, or dynamic adjustments to experiment parameters based on the results. The loop continues until the designated time range is exhausted, ensuring that each time slice is processed for optimal data handling and training efficiency.

This distributed approach effectively overcomes the limitations of the workflow reported previously^13^ by reducing memory bottlenecks and ensuring scalable data handling, with each rank processing its subset of the large dataset. This framework allows for seamless communication between the instrument and the computing resources, facilitating immediate adjustments to experimental parameters as the data are processed in real time. Once a pre-defined number of slices is accumulated, a templated job script is submitted to OLCF machines for both the TFT model training and the prediction of the data slice at the voxel level for future time steps. The relative error between the predicted data and the ground truth is logged and then checked by the main process to determine if the experiment has converged. The workflow allows for real-time data analysis and feedback during neutron scattering experiments, facilitating more efficient decision-making for experiment steering.

**Hardware** The main computation is performed on the Frontier supercomputer.^9,19,20^ Frontier consists of 9408 nodes, and each node is equipped with four AMD Instinct MI250X GPUs. All four MI250Xs (eight effective GPUs) are connected using 100 GB/s Infinity Fabric (200 GB/s between 2 GCDs of MI250X), and the nodes are connected via a Slingshot-11 interconnect with 100 GB/s of bandwidth. The data reduction is performed on the DGX workstation located at SNS, which is then streamed to OLCF via a 100 Gigabit Ethernet connection.

**Software** Our implementation stack is based on PyTorch v2.1 for TFT model training and Mantid v6.8.0 for data reduction (source code: https://code.ornl.gov/jqyin/topaz-tft and https://code.ornl.gov/vre/peak-integration).

## III. RESULTS AND DISCUSSION

This work successfully establishes an edge-to-exascale workflow for neutron scattering experiments, laying the groundwork for future developments in real-time experiment steering using live data streams. The key performance improvements achieved through this integration of edge computing, Frontier-based distributed training, and machine learning models, particularly the results from TFT, demonstrate the practical advantages of the workflow, such as reduced latency, increased computational efficiency. The integration of edge computing, Frontier-based distributed training, and machine learning models, particularly the TFT, demonstrates the practical advantages of the workflow, such as reduced latency and increased computational efficiency. These outcomes showcase the system's ability to handle large-scale neutron datasets with greater throughput, representing significant progress toward adaptive experiment steering and real-time data analysis at SNS.

### A. Data reduction at the edge

Data reduction is a crucial step in transforming TOF event data into a format that can be efficiently processed by machine learning models and high-performance HPC systems. To enable real-time steering of neutron scattering experiments, we developed a prototype application to predict volumetric neutron diffraction pattern in real time. This application was deployed on the DGX workstation at SNS, which can process either stored neutron event data or utilize ADARA for live data streaming, and feedback loop to EPICS for precise instrument control.

The primary goal of data reduction is to filter, map, and preprocess the event data, ensuring that only relevant neutron scattering events are selected for further analysis (Fig. 2). Given the high-dimensional nature of the data, reducing the data at the edge not only reduces the data load sent to the HPC system, but also significantly decreases latency in the feedback loop, enabling faster predictions and real-time experiment steering.

**FIG. 3.**
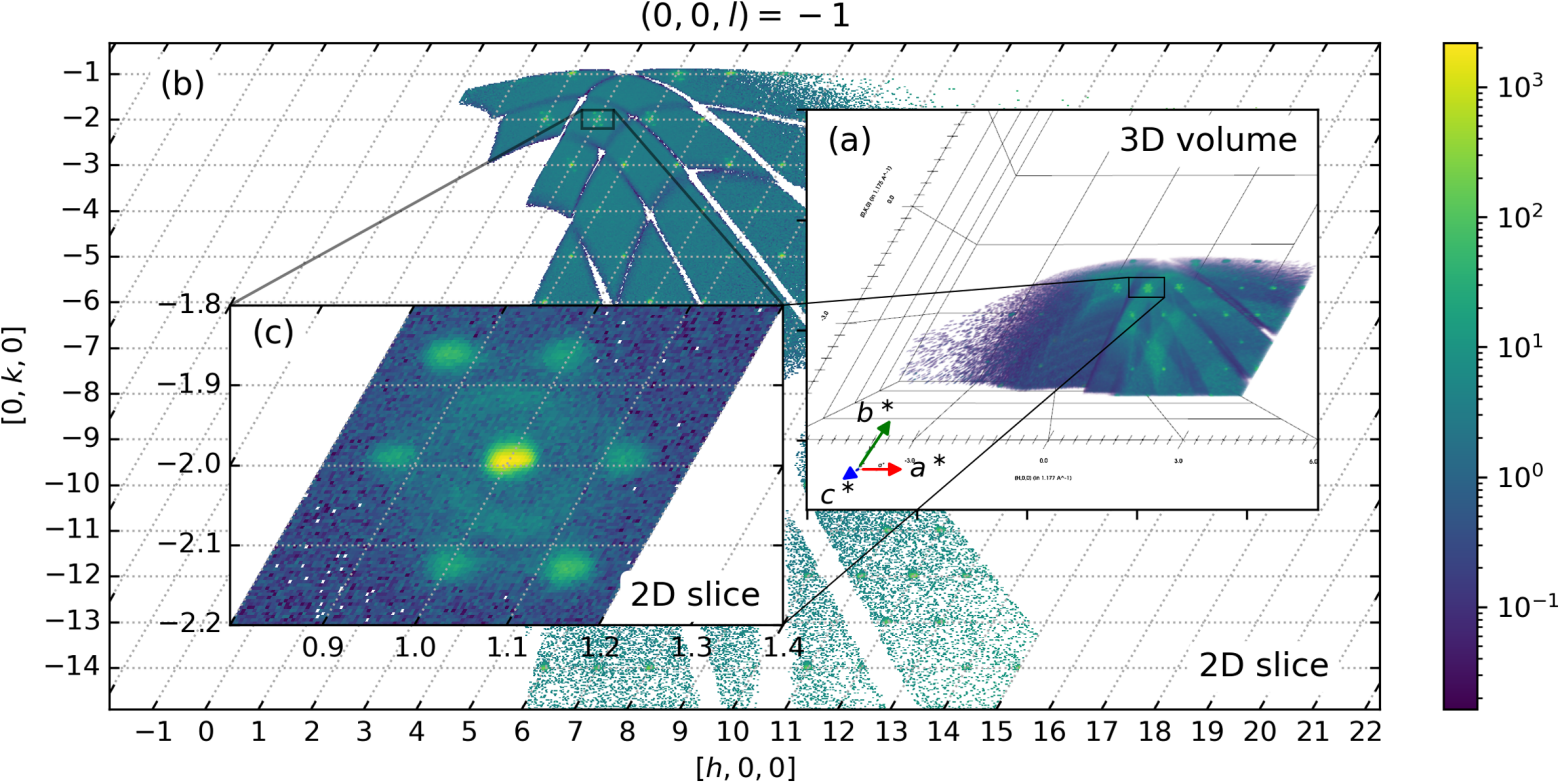
(a) 3D volume from a single time step, representing the complex, multi-dimensional neutron event data collected at SNS. The reciprocal lattice vectors a*, b*, and c* are drawn that represent the major non-orthogonal axes of the dataset and index diffraction spots with indices *h*, *k*, and *l*. (b) 2D slice from the volume of the (*h*, *k*, −1) plane revealing features such as peak locations and intensity modulations highlighted for real-time analysis. (c) The chosen 2D region of interest. This stepwise breakdown of data across dimensions highlights the efficiency of the edge-to-exascale framework, which divides large datasets by time steps and distributes them across ranks, enabling faster data processing and dynamic experiment steering.

Figure 3 illustrates key aspects of the real-time data generated by the edge-enabled system for data reduction during a single time step. The panel in Fig. 3(a) shows the 3D volume, capturing the complex multi-dimensional nature of neutron event data collected from the TOPAZ beamline at SNS. This 3D volume represents intensity distributions in neutron scattering patterns as a function of detector positions after a certain experiment time, providing essential insights into the underlying physical processes at play during the experiment. The 2D slice in Fig. 3(b) from one angular perspective is used for simplifying and interpreting the 3D signal. These slices help isolate and analyze specific features of the neutron scattering data, such as peak location, intensity distributions, and modulations shown in Fig. 3(c), that are critical for steering the experiment in real time.

This visual breakdown of the data across different dimensions is central to understanding how the edge-to-exascale framework accelerates both analysis and feedback loops during experiments. Dividing large datasets into files based on time steps and distributing them across ranks for preprocessing and training offers multiple advantages. In the previous workflow,^13^ loading and slicing the entire dataset at once placed significant memory burdens on a single rank, limiting scalability as data size grew. By implementing a new data loading scheme that handles data stepwise and distributes subsets across ranks, memory usage is optimized. Each rank processes smaller data chunks independently, reducing the memory footprint and enhancing parallelization. This approach accelerates preprocessing and training, avoids bottlenecks, and scales efficiently as the dataset expands, thereby ensuring the system remains responsive even under heavy data loads. By leveraging high-performance computing and edge-based data reduction, our system significantly decreases the time required to extract actionable insights from these complex datasets. The reduced latency between data acquisition and processing has been shown to enable experimenters to adjust parameters dynamically and optimize the outcomes within minutes instead of hours.

### B. Edge-to-exascale workflow efficiency

**FIG. 4.**
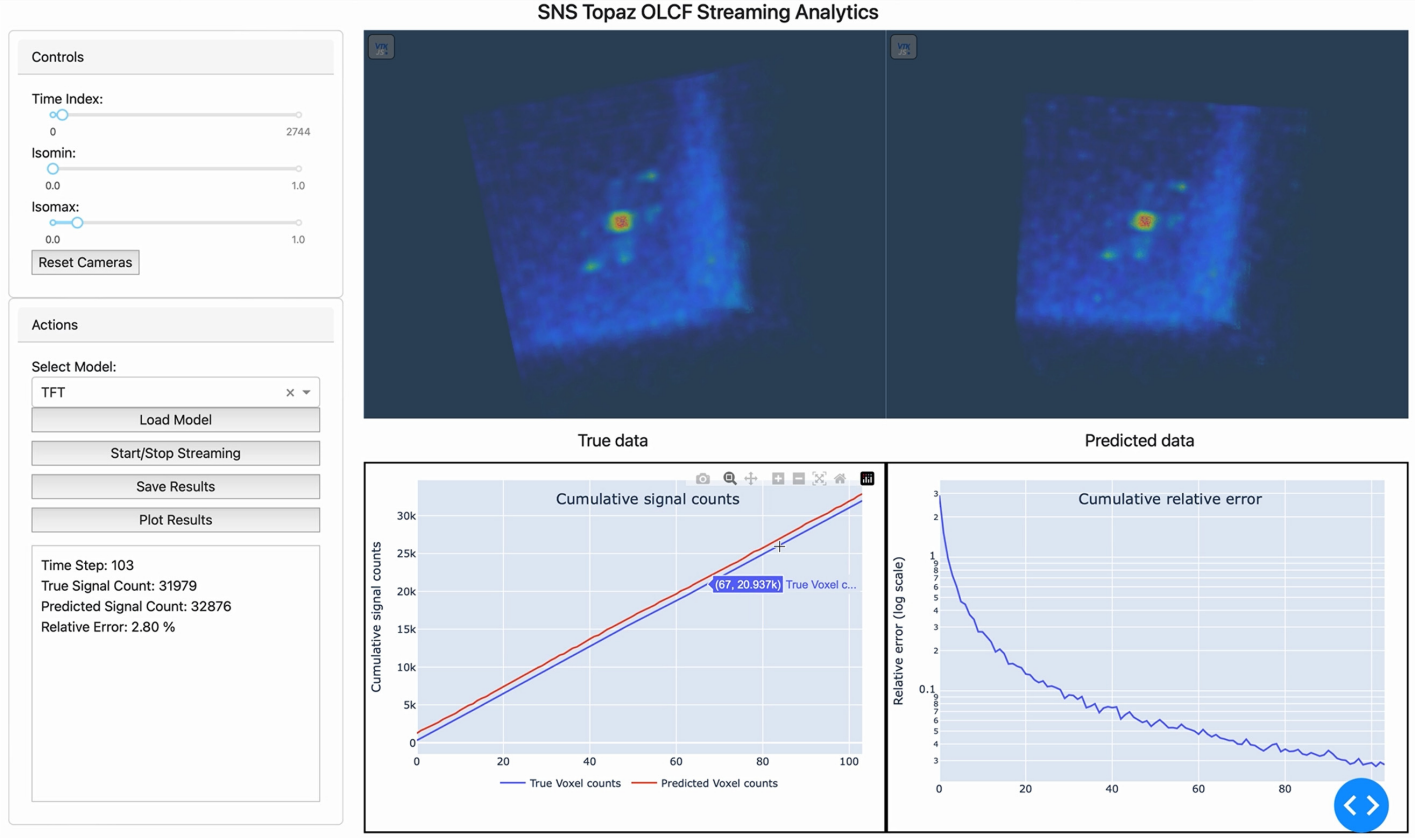
Graphic user interface for the edge-to-exascale workflow that links edge-based data processing on the DGX workstation with exascale computing resources (Frontier at OLCF). The upper panels show a comparison between true neutron scattering data (left) and predicted data from the TFT model (right), demonstrating prediction accuracy. The lower panels present cumulative signal counts (left) and relative error (right), with the system reporting a 2.8% relative error in real-time predictions. This interface enables researchers to make data-driven decisions during experiments, optimizing neutron beam time and improving experiment steering.

The integration of edge-based data processing and exascale computing enables efficient real-time steering of neutron scattering experiments. Figure 4 illustrates the graphic user interface (GUI) that links the edge-based data processing on the DGX workstation at the SNS with the exascale computing resources at Frontier, the world's first exascale computer, which is powered by AMD MI250x GPUs with HPE's Slingshot interconnect.^20^ Depending on the number of GPUs and input dimensions, the training of TFT can be completed within 10 min for 32 × 32 × 32 inputs using 512 GPUs (note that this only requires less than 1% of Frontier resources). In practice, we have tunable parameters (i.e., the number of voxels a GPU can process per dimension) to balance the trade-off between the resource and accuracy. This interface offers real-time feedback, allowing researchers to monitor the neutron scattering data as they are collected and processed, enhancing the experiment steering and decision-making capabilities.

The upper panels in Fig. 4 display visual comparisons between the true neutron scattering data (left) and predicted data (right), illustrating the accuracy of the machine learning models in forecasting neutron event distributions in real time. The alignment between the true and predicted neutron event distributions demonstrates the strength of our TFT model in accurately forecasting neutron event distributions across voxel space. The lower panels offer a more quantitative view, focusing on cumulative signal counts (left) and cumulative relative error (right), which give a direct indication of the model's performance throughout the experiment. This visual comparison helps users immediately assess model performance and adjust experimental parameters if necessary.

In the case presented in Fig. 4, the system reports a total signal count of 31 979 and a predicted signal count of 32 876, yielding a relative error of just 2.8%. These results fall well within the predefined convergence criteria of a relative error below 5%, underscoring the precision and reliability of the real-time predictions. Achieving such high accuracy not only improves the steering efficiency of the ongoing neutron scattering experiments but also optimizes the allocation of neutron beam time—an invaluable resource in high-demand user facilities like SNS. Without this advanced capability, traditional workflows would limit steering to broad adjustments, often based on incomplete or slowly processed data. This would lead to suboptimal use of beam time and experimental inefficiencies. In contrast, the edge-to-exascale workflow allows for fine-tuning experimental parameters based on real-time, data-driven insights. The integration of edge computing and exascale resources enables faster, more precise feedback loops, allowing researchers to make dynamic, data-driven decision throughout the experimental process. This not only boosts the productivity of experiments but also enhances the depth and quality of the scientific discoveries that can be made.

### C. Edge-to-exascale workflow for TOPAZ

The proposed edge-to-exascale workflow for neutron scattering experiments at SNS represents a significant advancement in data processing and real-time decision-making. This workflow leverages edge computing for real-time data preprocessing and transfers the processed data to exascale HPC environments, such as the Frontier supercomputer, for deeper analysis and model training. The focus of this section is to outline the key steps involved in this edge-to-exascale workflow, particularly in the context of the TOPAZ beamline, where the TFT model is utilized to predict neutron scattering patterns.

A key component of this workflow is the ability to integrate both stored 4D event data and live streaming of TOPAZ data via the ADARA system (Fig. 2). While NeXus files are used for detailed post-experiment analysis, ADARA provides live data streams optimized for real-time feedback and experiment steering. Both formats share similar metadata, but ADARA enables real-time processing of live data, allowing researchers to monitor experiments as they unfold. The TFT model can be applied to both stored and live data on HPC, ensuring flexibility and adaptability in a variety of experimental contexts.

The edge component of this workflow takes advantage of local computing resources, such as DGX workstations at SNS, to preprocess neutron event data before transferring it to Frontier. This preprocessing involves time filtering and slicing of 4D event data, effectively reducing the data volume and ensuring that only relevant information is passed to the exascale system.

Once the preprocessed data reach the Frontier supercomputer, the TFT model is deployed to predict neutron scattering patterns across multiple time horizons and 3D reciprocal space. With its multi-head attention mechanisms and variable selection capabilities, the TFT model can integrate diverse input features—such as neutron count rates, time-of-flight data, and spatial positions—to generate precise predictions. These predictions are then sent back to the edge for real-time experiment steering and decision-making. The ability of the TFT model to predict neutron event data over multiple timeframes is crucial for adaptive decision-making during time-of-flight experiments. Its attention mechanisms provide insights into dynamic neutron interactions, making it a powerful tool for guiding real-time experimental adjustments.

**FIG. 5.**
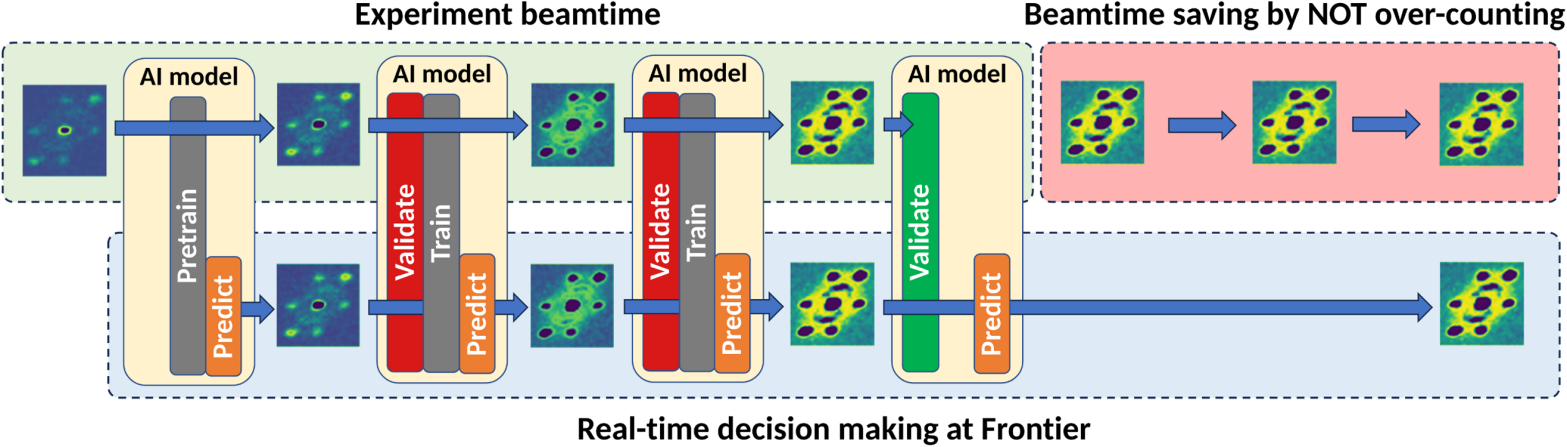
Schematic representation of real-time decision-making in neutron scattering experiments. Top: The traditional experimental workflow, which may result in overcounting and inefficient utilization of beam time. Bottom: A streamlined approach at Frontier, leveraging an AI model trained in real time with incoming experimental data. Data collection is halted as soon as the model's predictions align with the experimental results within a predefined accuracy threshold, enabling substantial savings in beam time.

Building upon our previously published work,^13^ which demonstrated the effectiveness of the TFT model for predicting neutron scattering patterns from 4D event data, we have integrated this capability into the edge-to-exascale workflow. This integration allows for dynamic adaptation of experiment durations based on real-time data analysis and model predictions derived from measured data. By utilizing edge computing, we have successfully minimized latency and streamlined data flow, both of which are essential for real-time feedback during experiments. Specifically, we achieved a 50% reduction in data processing latency and improvements of 22%–35% in ML model training time. Furthermore, the model accurately predicts neutron counts within a 128 × 128 × 128 block in HKL space surrounding a diffraction peak, achieving a relative error of less than 3.5% while using only 71% of the training data (Fig. 5). Such predictive capabilities enable the dynamic adjustment of experimental parameters during data collection, optimizing neutron beam time utilization and enhancing overall experimental efficiency.

To enable real-time feedback loop, we have streamlined the communication between the SNS data acquisition and analysis cluster and the Frontier supercomputer. Edge computing on a DGX workstation at SNS minimizes data latency and allows for preprocessing of live neutron event data. The preprocessed data are then transferred to Frontier via a data-pulling mechanism and automated job submission to the OLCF (Fig. 1). This streamlined workflow enables the TFT model to be rapidly trained on real-time data at the OLCF Frontier supercomputer and generate accurate predictions of neutron scattering patterns. The resulting framework, with its edge-enabled feedback loop, has the potential to transform how neutron scattering experiments are conducted at SNS. By enabling researchers to make rapid, data-driven adjustments, this workflow promises to optimize both time and resource utilization, leading to more precise and efficient analysis of multi-dimensional neutron event data and ultimately boosting beamline productivity.

This work establishes a robust foundation for the future of real-time decision-making in neutron scattering. While this workflow has not yet been fully deployed in a live experimental setting, it demonstrated capabilities and proof-of-principle validation pave the way for further advancements in experimental methodologies and hold the promise of significantly enhancing the efficiency and scientific output of neutron scattering facilities. The application is built with portability to the Neutron Data Interpretation Platform (NDIP) to enable seamless scalability across different beamlines using ADARA data streams. Our evaluations, based on pre-defined convergence criteria (e.g., relative error < 5%), showed great potential for high accuracy in real-time predictions, with a potential to save up to 29% of neutron beam time—contributing to a more efficient neutron resource utilization and reduced operational costs. These results, based on a single proof-of-concept experiment, validate the workflow's feasibility. Future work will test its generalizability across multiple experiments and provide additional statistical metrics.

### D. Analysis of predicted data

**FIG. 6.**
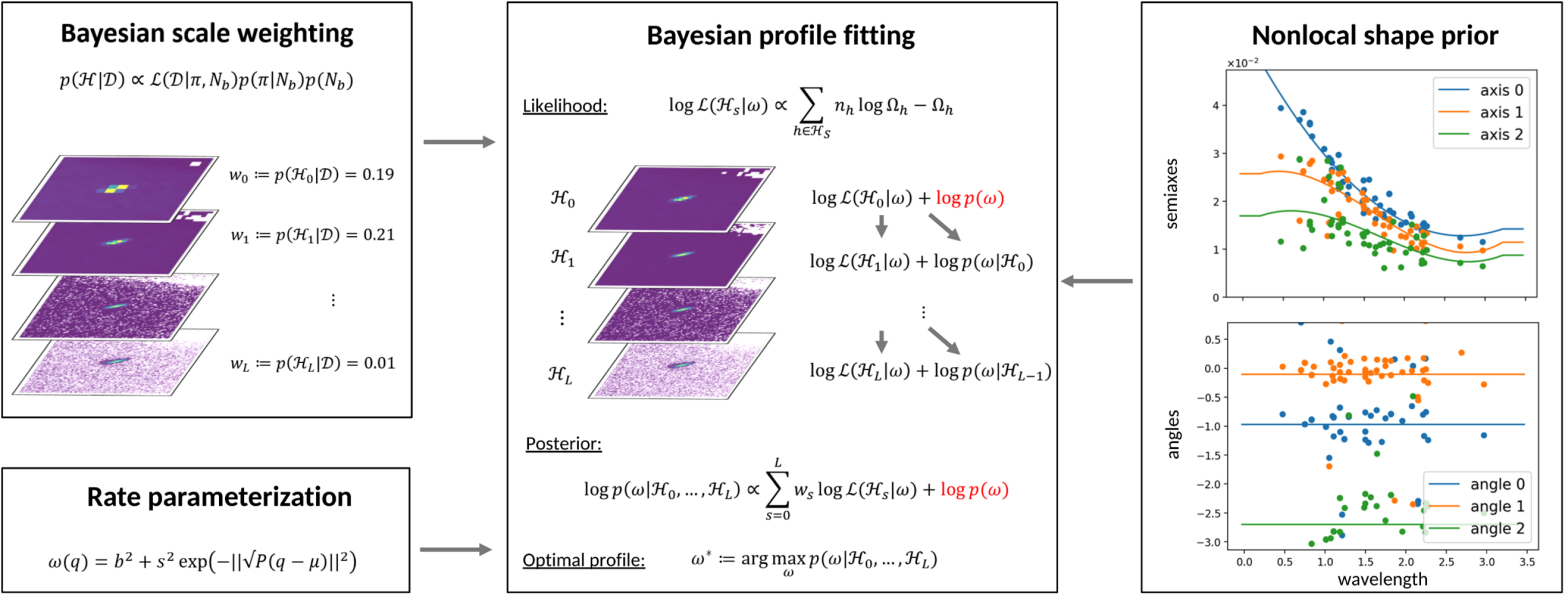
Schematic illustration of the Bayesian profile fitting approach for adaptive Bragg peak integration. The method combines Bayesian scale weighting, rate parameterization, and a nonlocal shape prior to optimize peak shape and intensity estimation. It uses hierarchical likelihoods and nonlocal priors to improve peak shape estimation, especially in low signal-to-noise regions.

The proposed edge-to-exascale workflow leverages streaming event data to forecast the progression of data collection. Predictions from the TFT model at Frontier are validated at the edge to provide feedback for real-time decision-making and experimental steering (Fig. 5). This validation process involves monitoring standard voxel-wise error metrics in 3D HKL space and identifying discrepancies in calculated peak intensities, which are critical for crystal structure refinement. A key challenge for this workflow is determining meaningful peak intensities early in data collection, when much of the data remains under-resolved due to low signal-to-noise ratios. Traditional methods are poorly suited to this task, as they depend on uniformly well-resolved data, which is often unavailable during initial stages. Instead, we accomplish this task with a hierarchical Bayesian approach for adaptive integration of Bragg peaks (Fig. 6).^21^

Our approach features scale space representation of data in reciprocal coordinates that relies on exploiting intrinsic data redundancies to “fill the gaps” in regions with low event counts. Mathematically, this is accomplished by solving an optimization problem with the hierarchical likelihood over a sequence of histograms and the nonlocal prior utilizing shape similarities of different peaks. The likelihood encodes statistical assumptions on the data generating process and drives the optimization problem to determine the neutron rate that is most likely to produce the observed data. The hierarchical construction is used to mitigate the challenge of representative histogram scale selection and to ensure the consistency of peak shape estimation in the process of sequential data collection. Similarly, the nonlocal prior is used to facilitate the shape estimation of weaker peaks by relating them to stronger peaks via the dependence of peak shapes on physical parameters such as the wavelength.

**FIG. 7.**
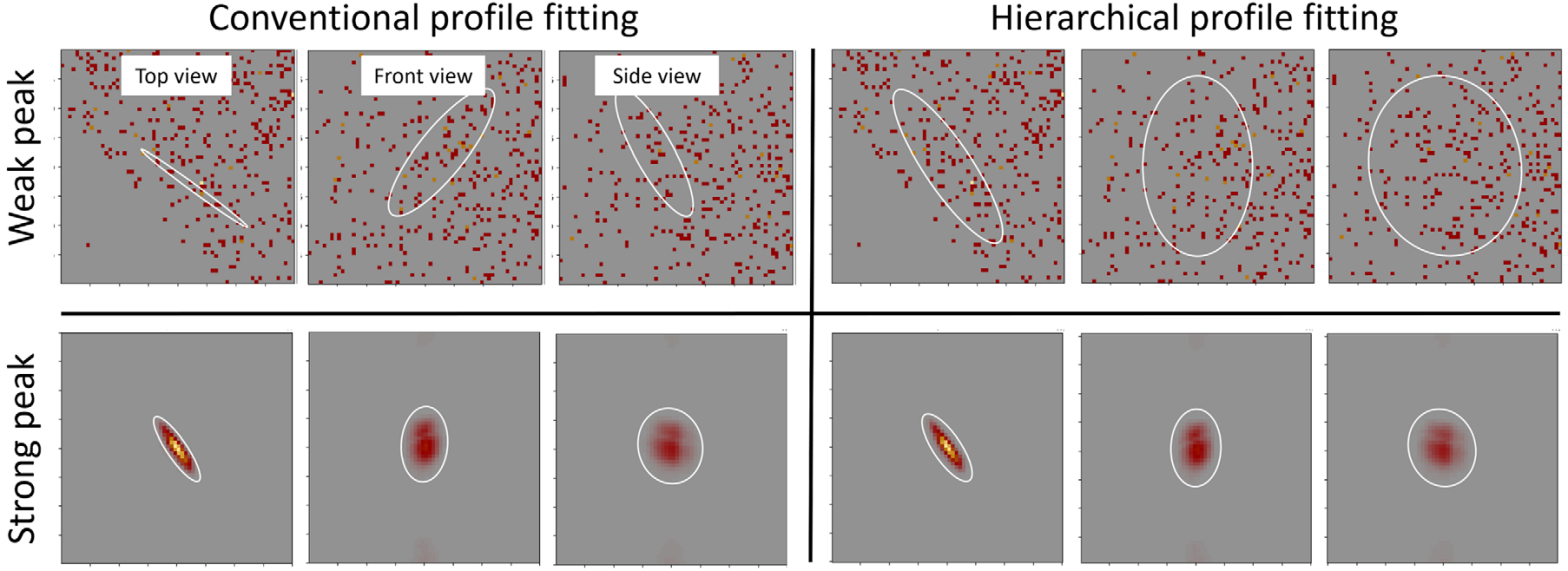
Estimated peak shapes using both conventional and proposed approaches. The hierarchical fit with a nonlocal prior produces consistent shapes for both strong and weak peaks, while the conventional approach shows instability and significant variability, leading to larger fluctuations in peak intensities.

Figure 7 illustrates the result of the proposed peak estimation approach in comparison with the conventional profile fitting of a single isolated peak. While strong peaks are reliably estimated in both methods, weak peaks pose challenges for the conventional approach, leading to unstable fits and high variability that result in significant fluctuations in peak intensities (top left). These fluctuations undermine the accuracy and consistency required to validate the TFT model predictions from hierarchical profile fitting (top right), as conventional profile fitting introduces variability that can obscure meaningful patterns and reduce confidence in the model's ability to accurately forecast neutron scattering behavior. By ensuring more consistent peak intensity estimates, the proposed method establishes a solid foundation for accurate TFT model validation. Once validated, the model aids in refining the estimated constant background, enabling precise calculation of peak intensities for downstream crystal structure refinement, ultimately improving the reliability of structural insights derived from the data.

### E. Impact on neutron user program at ORNL

The benefits of the edge-to-exascale workflow extend beyond individual experiments to the broader ORNL Neutron Program. By enhancing experiment steering efficiency and reducing resource consumption, our system improves the overall productivity of neutron beamlines. Researchers can now maximize their beam time outputs by making on-the-fly decisions based on real-time data analysis, such as the values of *R*_sig_, the ratios of the standard deviations to the measured or predicted peak intensities. This optimization not only accelerates the discovery process but also makes neutron beamlines more accessible to a larger number of users, fostering greater scientific collaboration and innovation within the neutron scattering community. The proof-of-principle workflow on the TOPAZ beamline serves as a blueprint for its implementation across other instruments at SNS and beyond. Future advancements, such as integrating dynamic feedback loops and enabling real-time responses to external stimuli like temperature, pressure, or magnetic fields, have the potential to further optimize beam time utilization and improve experimental outcomes. These developments will enhance the accessibility and productivity of neutron beamlines, supporting broader scientific collaboration and innovation within the ORNL Neutron User Program.

## IV. SUMMARY

We have developed an edge-to-exascale workflow aimed at enhancing neutron scattering experiments through the integration of advanced machine learning (ML), artificial intelligence (AI), and high-performance computing (HPC) methods. Building on previous research with the Temporal Fusion Transformer (TFT) model for predicting neutron scattering patterns from 4D event data, this framework moves closer to enabling real-time analysis and feedback. The combination of the TFT model with the edge-to-HPC approach allows for forecasting neutron event data streams, significantly reducing latency in data analysis. The system demonstrates that adaptive adjustments based on predicted data can enhance beam time efficiency, improve data quality, and optimize experimental outcomes. The ML algorithm could help reduce the over-counting of neutron beamtime by around 29% at TOPAZ, while achieving the similar data quality. These findings highlight the transformative potential of integrating scalable AI techniques across a wide range of neutron scattering experiments. Building on this foundation, this approach not only supports future advancements in real-time experiment steering but also underscores the broader applicability of scalable AI in neutron data analysis. Future efforts will focus on refining the integration of ML models and edge computing to enable fully automated real-time steering. A key priority will be the development of live data integration capabilities, empowering researchers to dynamically adjust experimental conditions in real time, maximizing beam time efficiency and driving new scientific discoveries. Further extending this vision, plans include exploring dynamic feedback loops to facilitate real-time responses to external stimuli, such as temperature, pressure, electric, or magnetic fields, further advancing adaptability and enabling adaptive data collection strategies to achieve full automation of neutron scattering experiments.

## Data Availability

The data that support the findings of this study are available from the corresponding author upon reasonable request.
